# Supplementary material for: Analysis of the intracellular traffic of IgG in the context of Down syndrome (trisomy 21)
Source: Sci Rep. 2021 May 26;11:10981. doi: 10.1038/s41598-021-90469-z (PMC8155081; doi:10.1038/s41598-021-90469-z)
Supplement: Supplementary file 1 — Supplementary Information 1. [file 41598_2021_90469_MOESM1_ESM.pdf]

Supplemental Information

Analysis of the intracellular traffic of IgG in the context of Down syndrome (trisomy 21).

Cejas RB[1], Tamaño-Blanco M[1], and Blanco JG[1,*]

[1] Department of Pharmaceutical Sciences, School of Pharmacy and Pharmaceutical Sciences, The State University of New York at Buffalo, Buffalo, NY 14214, USA

* Corresponding author at: Department of Pharmaceutical Sciences, University at Buffalo, The State University of New York, 470 Pharmacy building, Buffalo, NY 14214 – 8033, USA

Table S1. Human fibroblasts

ID	Catalog #	Description	Age	Gender
NDS-1	Coriell Cat# GM08680, RRID:CVCL_7489	Healthy	5 months	Male
NDS-2	Coriell Cat# AG07095, RRID:CVCL_0N66	Healthy	2 years	Male
NDS-3	Coriell Cat# GM03234, RRID:CVCL_9W93	Healthy	21 years	Male
NDS-4	Coriell Cat# GM00023, RRID:CVCL_7268	Healthy	31 years	Female
DS-1	Coriell Cat# AG07096, RRID:CVCL_X868	Trisomy 21	5 months	Male
DS-2	Coriell Cat# AG06922, RRID:CVCL_X793	Trisomy 21	2 years	Male
DS-3	Coriell Cat# GM01920, RRID:CVCL_V464	Trisomy 21	21 years	Male
DS-4	Coriell Cat# GM02767, RRID:CVCL_V469	Trisomy 21	14 years	Female

Table S2. PCR primers and siRNA

**qRT-PCR primers**	**Primer forward sequence 5’→3’**	**Primer reverse sequence 5’→3’**
qRT-PCR-*FCGRT*	TCGTGGTGGGAATCGTC	CACGAAGGGAGATCCAAGGG
qRT-PCR-APP	CATCATGGTGTGGTGGAGGTTGA	CTGTGGCGGGGGTCTAGTT
qRT-PCR-B-Actin	GGACTTCGAGCAAGAGATGG	AGCACTGTGTTGGCGTACAG
**siRNA**	**Sense Strand 5’→3’**	**Anti-sense Strand 5’→3’**
FCGRT siRNA	CUGUUUUCCACCUCGAUAAUU	UUAUCGAGGUGGAAAACAGUU

Table S3. *APP* mRNA relative fold expression in human fibroblasts

ID	Catalog #	Description	*APP* (relative fold ± SD)
NDS-1	GM08680	Healthy	1.44 ± 0.21
NDS-2	AG07095	Healthy	0.83 ± 0.14
NDS-3	GM03234	Healthy	0.65 ± 0.06
NDS-4	GM00023	Healthy	1.08 ± 0.26
DS-1	AG07096	Trisomy 21	1.57 ± 0.16
DS-2	AG06922	Trisomy 21	1.38 ± 0.21
DS-3	GM01920	Trisomy 21	2.16 ± 0.75
DS-4	GM02767	Trisomy 21	3.43 ± 0.62

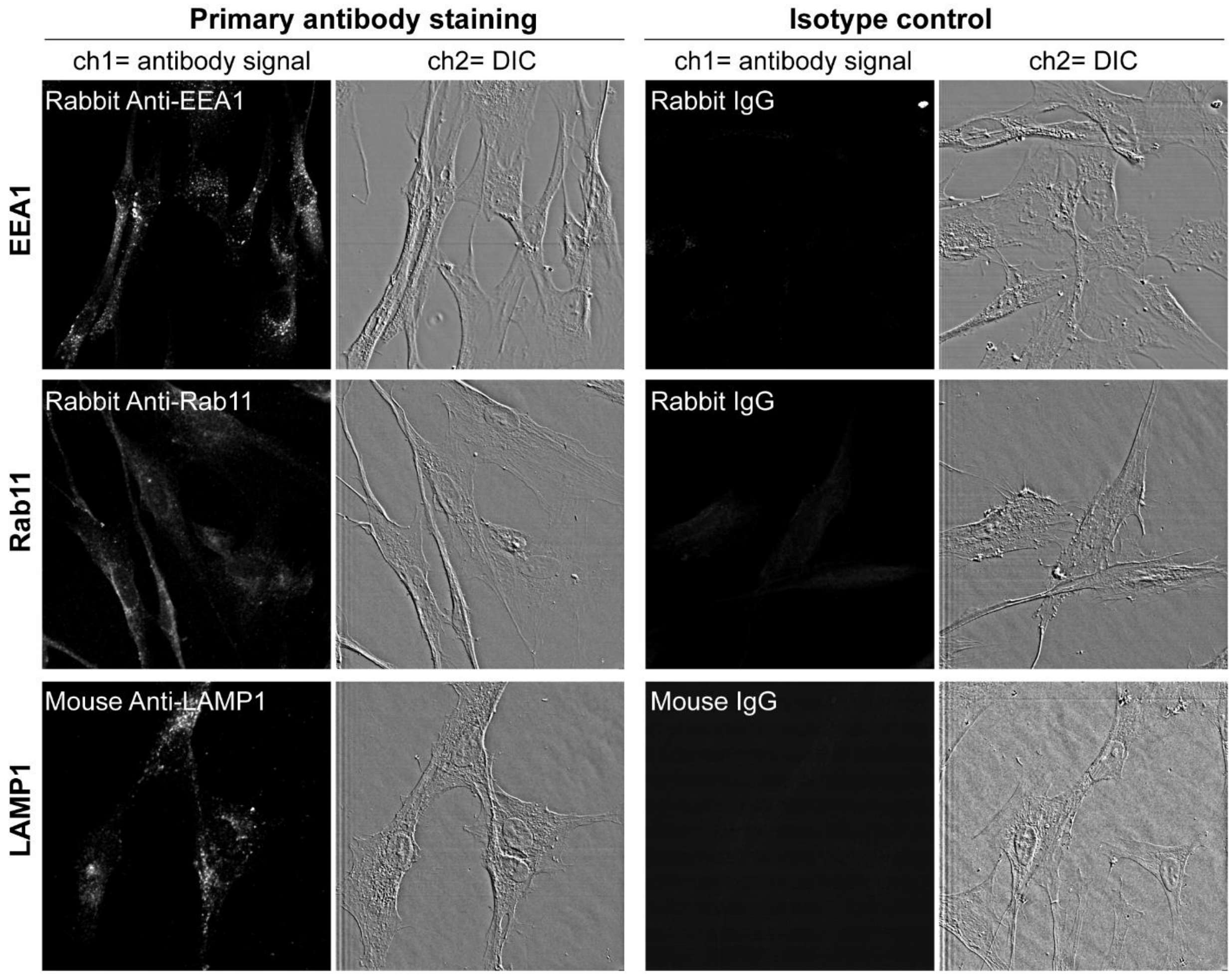

Figure S1. Expression of endosomal markers in fibroblasts. EEA1, Rab11 and LAMP1 expression detected with specific antibodies and corresponding IgG isotype controls for the analysis of immunostaining specificity under identical imaging conditions. Cell borders were detected in the differential interference contrast (DIC) channel.

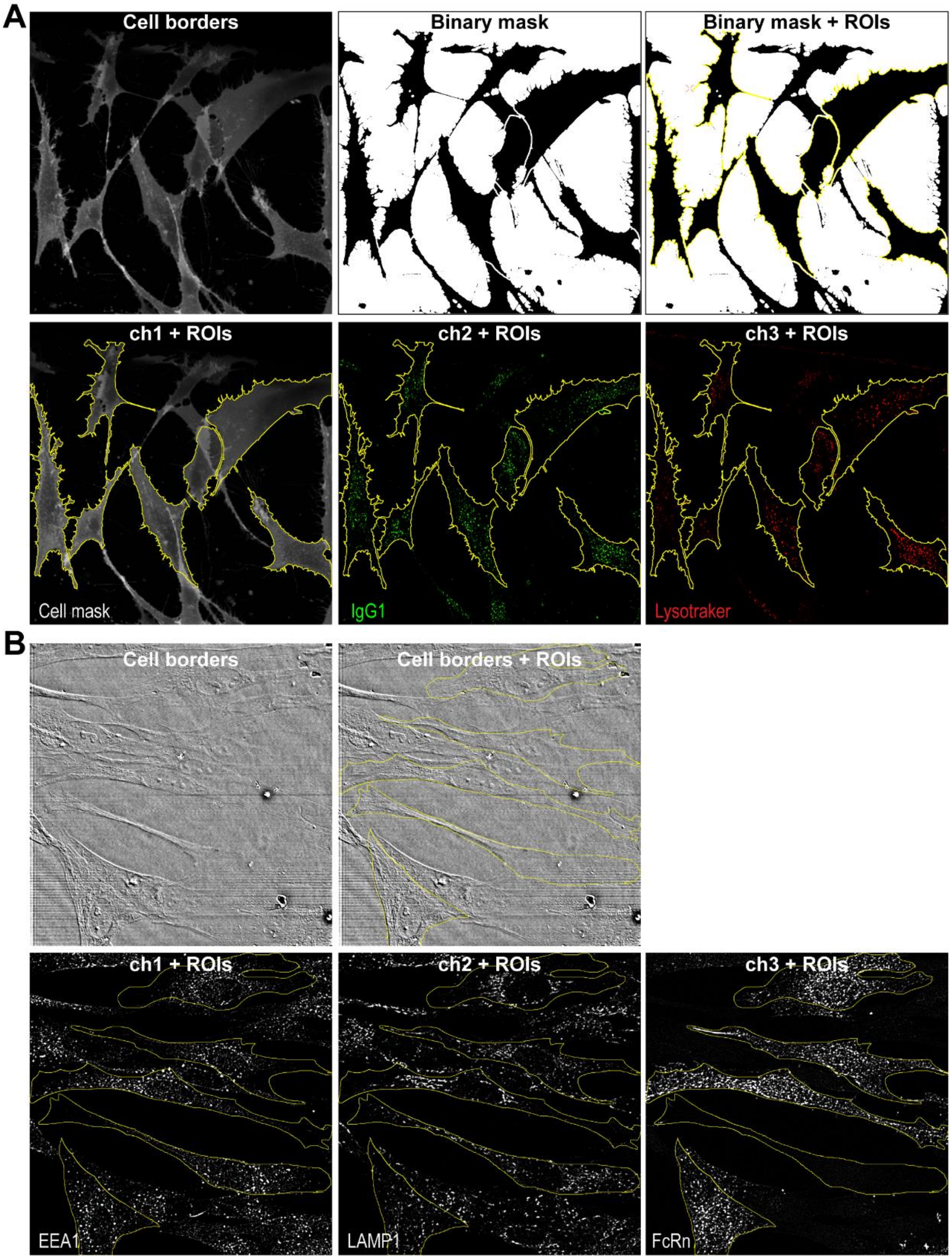

Figure S2. Generation of cellular regions of interest for quantitative image analysis. Representative fields of view showing regions of interest (ROIs, yellow) corresponding to individual cells created by segmentation from CellMask plasma membrane stain channel **(A)** or differential interference contrast/bright field channel **(B)**. For panels A and B, images at the top show ROIs generated from cell borders, and images at the bottom show the vesicles within ROIs that were included for quantification.

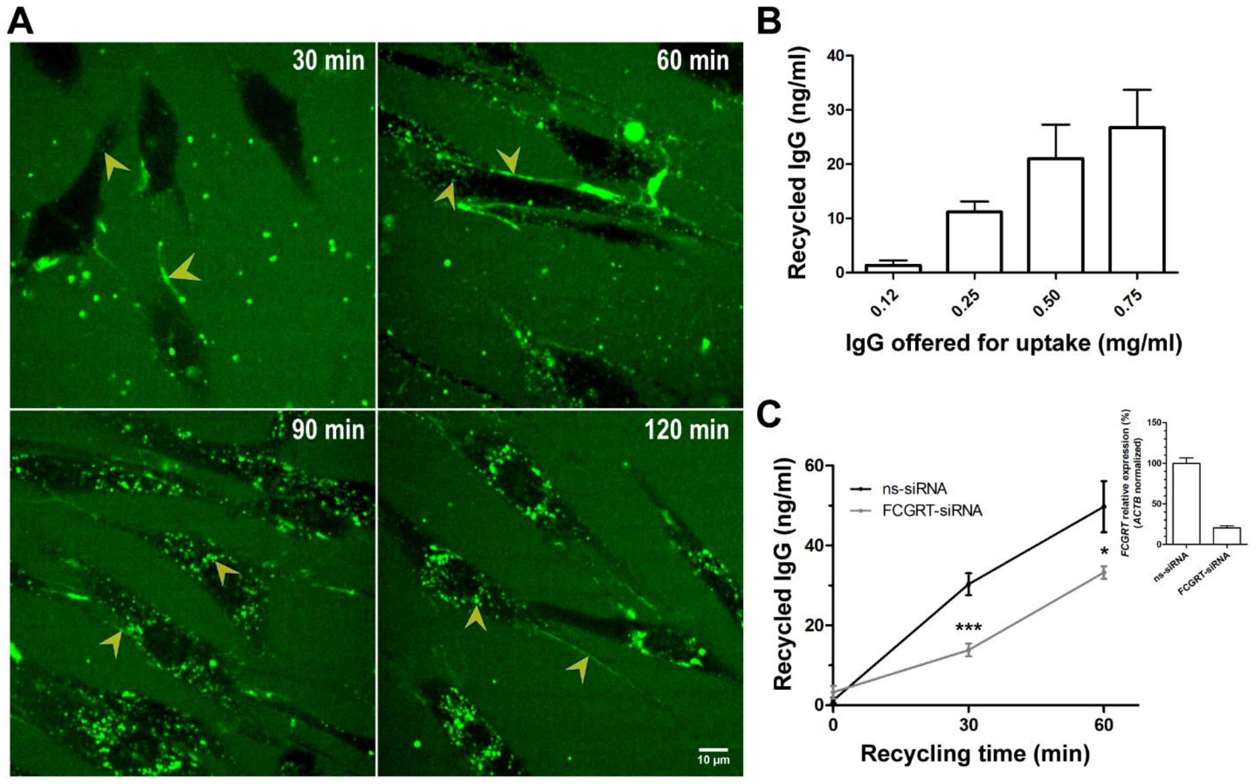

Figure S3. IgG1 uptake and recycling screening in human fibroblasts. A. Live cell imaging of IgG (green) uptake after incubation with 0.25 mg/ml Alexa conjugated-hIgG1 for 30, 60, 90, or 120 min at 37 °C. Yellow arrows show IgG distribution in cell membrane and intracellular vesicles. Scale Bar: 10 µm. **B.** IgG concentration in cell media after 60 min of recycling at 37 °C in cells previously incubated for 60 min with variable concentrations of hIgG1 for uptake. Each bar represents the mean ± SD from two measurements performed in duplicates. **C.** IgG concentration in media of transfected cells after 0, 30, or 60 min of recycling and previous incubation for uptake with 0.50 mg/ml hIgG1 for 60 min. Cells were transfected with a non-sense siRNA (ns-siRNA, control) or siRNA against *FCGRT* (*FCGRT*-siRNA). Each point represents the mean ± SEM from two measurements performed in duplicates. *** $P < 0.001$, * $P < 0.05$, Mann Whitney test.

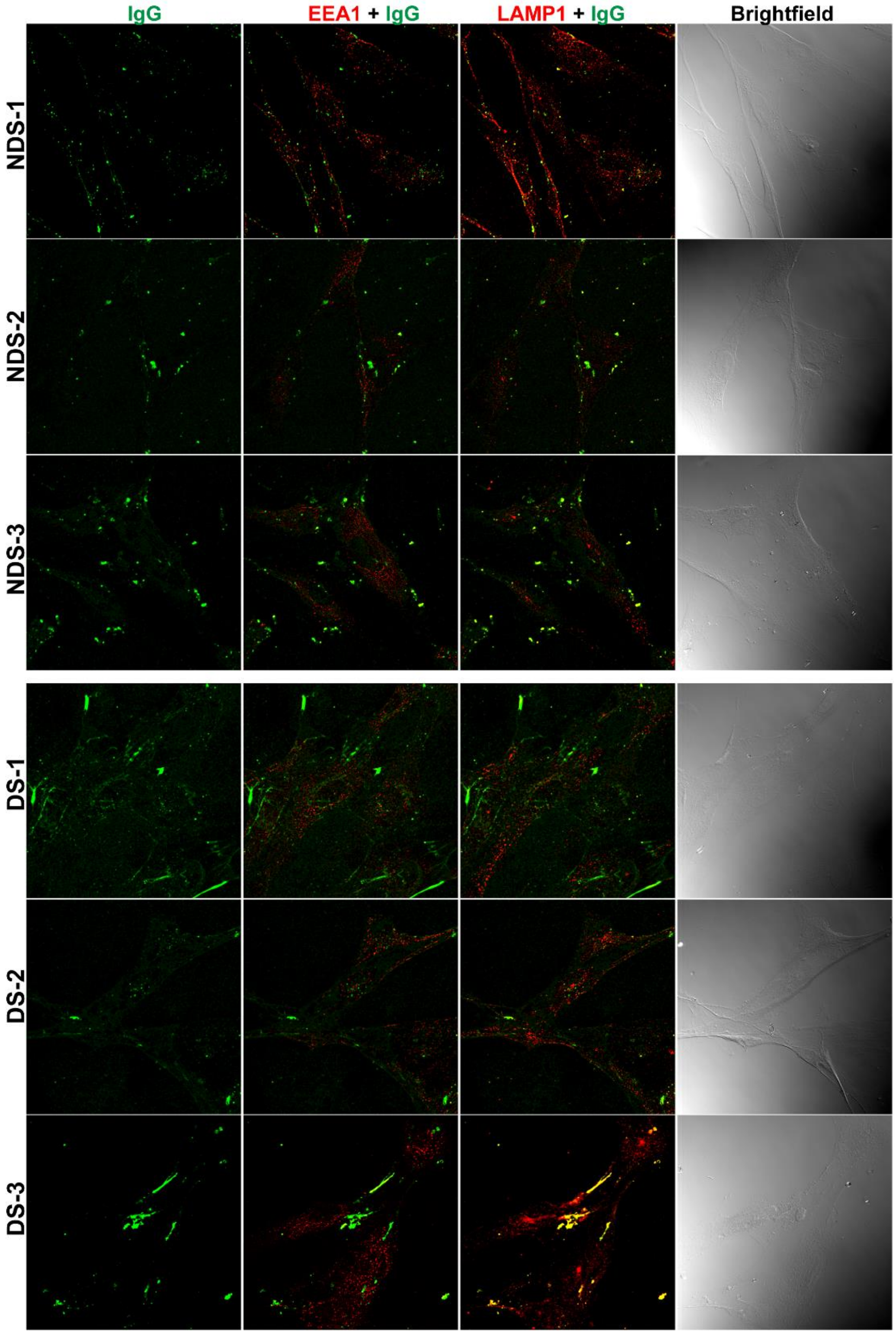

Figure S4. IgG1 uptake in fixed cells. Representative images of diploid (NDS-1, NDS-2, and NDS-3) and trisomic (DS-1, DS-2, and DS-3) human fibroblasts. After incubation with 0.50 mg/ml hIgG1 for 60 min at 37 °C, cells were fixed, permeabilized, and immunostained with anti-human IgG (green), anti-EEA1 (red) or anti-LAMP1 (red). Cell borders were detected with brightfield. Note that virtual LUTs are employed, and merged images are shown as green/red to facilitate visualization of overlapping signals.

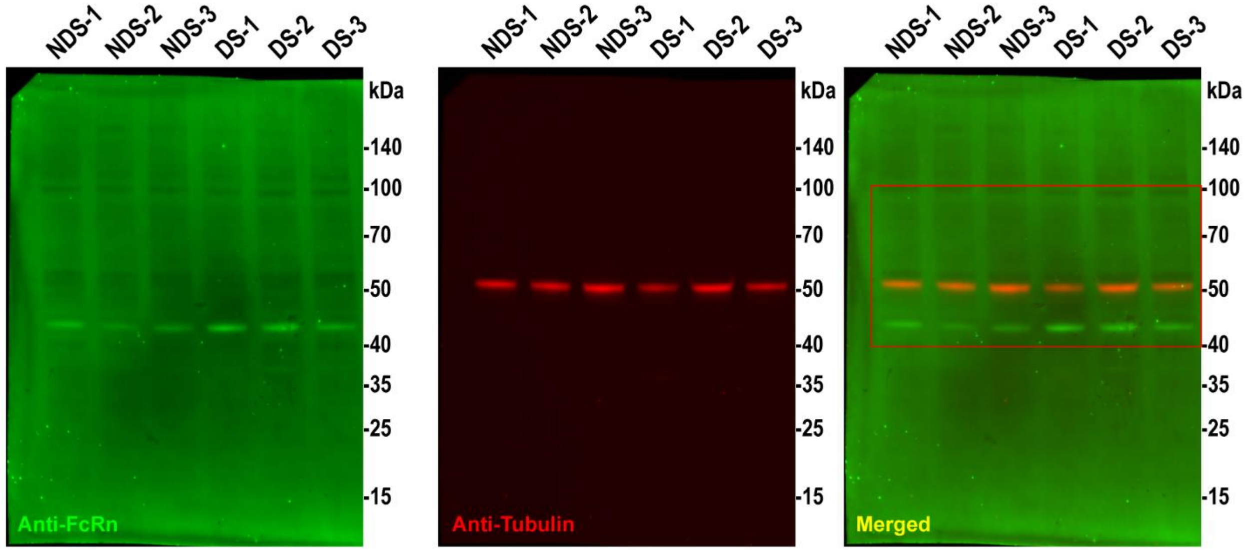

Figure S5. FcRn expression in diploid and trisomic cells. FcRn expression detected by immunoblotting with an anti FcRn antibody (green) in diploid (NDS-1, NDS-2, and NDS-3) and trisomic (DS-1, DS-2, and DS-3) fibroblasts. Tubulin (red) was assayed as loading control. Red box indicates the cropped region shown in Figure 5, panel A.

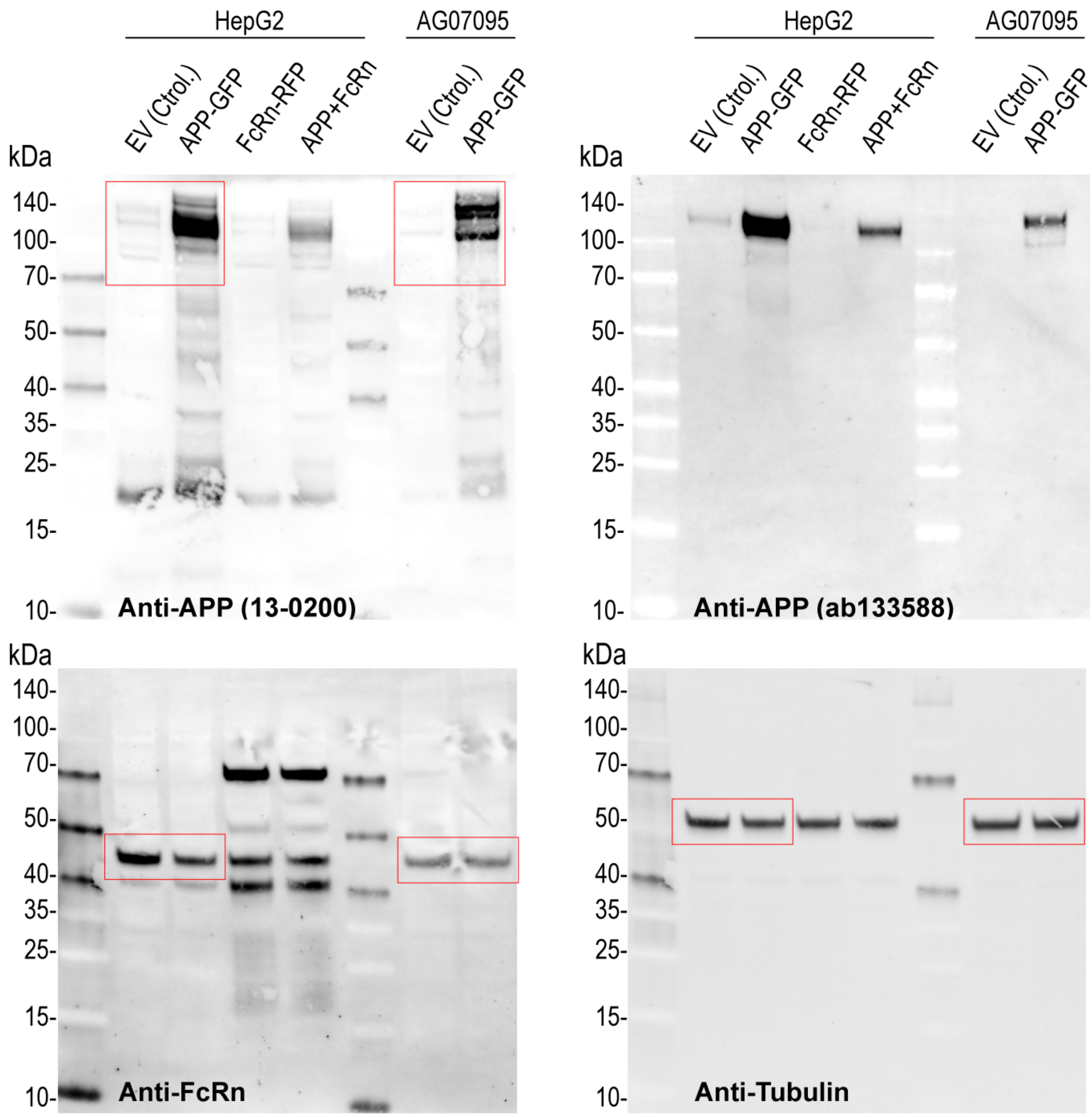

Figure S6. APP and FcRn expression in diploid cells. APP and FcRn expression detected by immunoblotting with two anti-APP antibodies (13-0200 from Invitrogen and ab133588 from Abcam) and anti FcRn in AG07095 and HepG2 cells. Proteins were extracted after 48 h of transfection with an empty vector (EV, Control), or plasmids that codify for APP-GFP, or FcRn-RFP. Tubulin was assayed as loading control. Red boxes indicate cropped regions shown in Figure 5, panels D (AG07095 cell line, right boxes on blots) and E (HepG2 cell line, left boxes on blots).

A

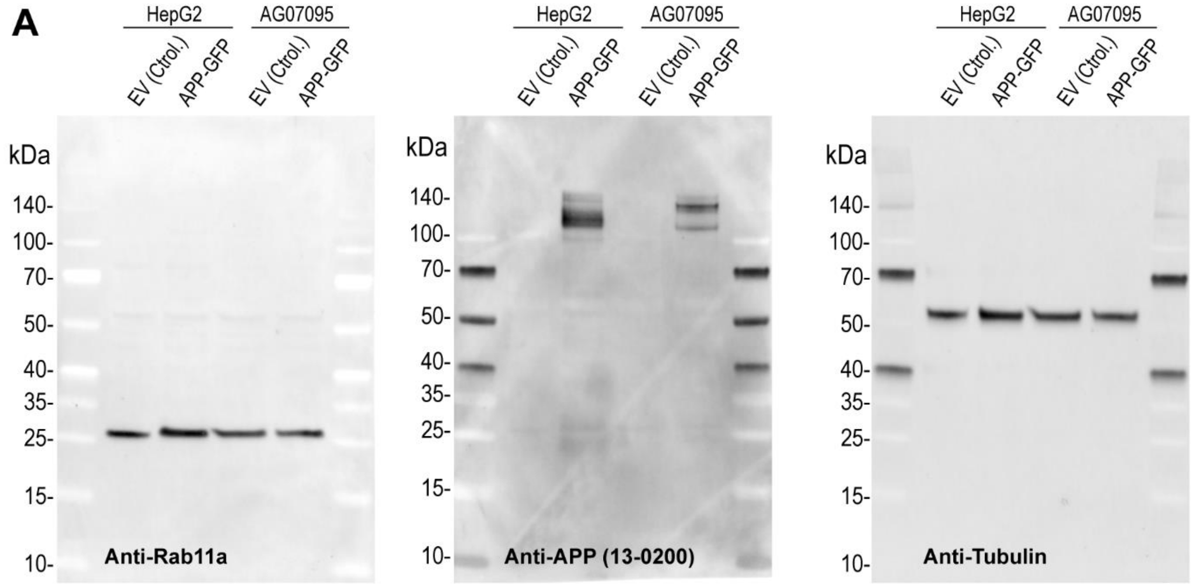

B

HepG2

Rab11 (normalized)	
**EV**	**APP-GFP**
1.00	1.01

AG07095

Rab11 (normalized)	
**EV**	**APP-GFP**
1.00	1.07

Figure S7. Rab11 expression in diploid cells. A. Rab11 expression detected by immunoblotting with anti-Rab11 antibody in AG07095 and HepG2 cells. Proteins were extracted after 48 h of transfection with an empty vector (EV, control), or a plasmid that codify for APP-GFP. APP expression was assessed with an anti-APP antibody (13-0200 from Invitrogen). Tubulin was assayed as loading control. **B.** Densitometric analysis of Rab11 expression, normalized to tubulin.

Video 1. Intracellular transport of IgG (green) in human fibroblasts (AG06922 cells), after incubation with 0.25 mg/ml Alexa Fluor 633-IgG1 for 60 min at 37 °C. Lysosomes were stained with Lysotracker (red). Yellow regions show IgG distributed in the degradative pathway. The time lapse stack is composed by 80 frames, imaged in a frame interval of 7.5 sec/frame.
